# Supplementary material for: The Link Between Oxysterols and Gut Microbiota in the Co-Dysfunction of Cognition and Muscle
Source: Nutrients. 2025 Apr 6;17(7):1277. doi: 10.3390/nu17071277 (PMC11990608; doi:10.3390/nu17071277)
Supplement: Supplementary file 1 [file nutrients-17-01277-s001.zip › nutrients-3561666-supplementary.pdf]

S1. Composition of Food Frequency Questionnaire

Table S1 Composition of Food Frequency Questionnaire

Food type	frequency of eating				Average weight per serving (50g)
	Day	Week	Month	Year	
1. rice					
2. wheat flour					
3. coarse cereals					
4. potatoes and yam					
5. fried pasta					
6. pork					
7. beef					
8. mutton					
9. chicken					
10. duck					
11. donkey meat					
12. visceral food					
13. other meat					
14. aquatic product					
15. milk					
16. milk powder					
17. cheese					
18. yogurt					
19. eggs					
20. tofu					
21. silk tofu					
22. tofu curd					
23. dried bean curd					
24. soya milk					
25. dried beans					
26. fresh vegetables					
27. dried vegetable					
28. salted vegetables					
29. pickles					
30. Chinese sauerkraut					
31. fresh fruit					
32. snack					
33. drinks					
Extra: cooking oil					

S2. The step-by-step detailed protocol for ELISA kit

(1) APP

① Bring all reagents to room temperature (18-25℃) before use. Dilute 30 mL of Concentrated Wash Buffer with 720 mL of deionized or distilled water to prepare 750 mL of Wash Buffer.

② Centrifuge the standard at 10,000×g for 1 min. Add 1 mL of Reference Standard &Sample Diluent, let it stand for 1-2 min and then mix it thoroughly with a vortex meter of low speed. Then make serial dilutions as needed. The recommended dilution gradient is as follows: 5000, 2500, 1250, 625, 312.5, 156.25, 78.13, 0 pg/mL.

③ Biotinylated Detection Ab working solution/HRP Conjugate working solution: Centrifuge the Concentrated Biotinylated Detection Ab/HRP Conjugate working solution at 800×g for 1 min, then dilute the 100× Concentrated Biotinylated Detection Ab to 1× working solution with Biotinylated Detection Ab Diluent. The working solution should be prepared just before use.

④ Determine wells for diluted standard, blank and sample. Add 100 μL each dilution of standard, blank and sample into the appropriate wells. Cover the plate with the sealer provided in the kit. Incubate for 90 min at 37°C.

⑤ Decant the liquid from each well, do not wash. Immediately add 100 μL of Biotinylated Detection Ab working solution to each well. Cover the plate with a new sealer. Incubate for 1 hour at 37°C.

⑥ Decant the solution from each well, add 350 μL of wash buffer to each well. Soak for 1 min and aspirate or decant the solution from each well and pat it dry against clean absorbent paper. Repeat this wash step 3 times.

⑦ Add 100 μL of HRP Conjugate working solution to each well. Cover the plate with a new sealer. Incubate for 30 min at 37°C.

⑧ Decant the solution from each well, repeat the wash process 5 times as conducted in step ⑥.

⑨ Add 90 μL of Substrate Reagent to each well. Cover the plate with a new sealer. Incubate for about 15 min at 37°C. Protect the plate from light.

⑩ Add 50 μL of Stop Solution to each well. Note: adding the stop solution should be done in the same order as the substrate solution.

⑪ Determine the optical density (OD value) of each well at once with a micro-plate

reader set to 450 nm.

⑫ Average the readings for each standard and samples, then subtract the average zero standard optical density. Plot a four-parameter logistic curve on log-log axis, with standard concentration on the x-axis and OD values on the y-axis.

(2) Aβ1-42

Except for the recommended dilution gradient, the other steps are the same as those of APP.

The recommended dilution gradient for Aβ1-42 detection: 1000、500、250、125、62.5、31.25、15.63、0 pg/mL

(3) Nfl

Except for the recommended dilution gradient, the other steps are the same as those of APP.

The recommended dilution gradient for Nfl detection: 1000、500、250、125、62.5、31.25、15.63、0 pg/mL.

(4) CAF

Except for the following steps, the other steps are the same as those of APP.

② The recommended dilution gradient for CAF detection: 2000、1000、500、250、125、62.5、31.25、0 pg/mL.

④ Incubate the plate for 80 min at 37°C.

⑤ Decant the liquid from each well, add 200 μL of wash buffer to each well. Soak for 1~2 min and aspirate or decant the solution from each well and pat it dry against clean absorbent paper. Repeat this wash step 3 times. Immediately add 100 μL of Biotinylated Detection Ab working solution to each well. Cover the plate with a new sealer. Incubate for 50 min at 37°C.

⑥ Decant the solution from each well, add 200 μL of wash buffer to each well. Soak for 1~2 min and aspirate or decant the solution from each well and pat it dry against clean absorbent paper. Repeat this wash step 3 times.

⑦ Add 100 μL of HRP Conjugate working solution to each well. Cover the plate with a new sealer. Incubate for 50 min at 37°C.

(5) Irisin

Except for the serum dilution multiple, the other steps are the same as those of APP.

Dilution ratio for serum irisin detection: 50 times

(6) BDNF

Except for the recommended dilution gradient and the serum dilution multiple, the other steps are the same as those of APP.

The recommended dilution gradient for BDNF detection: 2000、1000、500、250、125、62.5、31.25、0 pg/mL.

Dilution ratio for serum BDNF detection: 50 times.
